# Robustness of Brain Structural Networks Is Affected in Cognitively Impaired MS Patients

**DOI:** 10.3389/fneur.2020.606478

**Published:** 2020-11-19

**Authors:** Hamza Farooq, Christophe Lenglet, Flavia Nelson

**Affiliations:** ^1^Department of Neurology, University of Minnesota, Minneapolis, MN, United States; ^2^Center for Magnetic Resonance Research, University of Minnesota, Minneapolis, MN, United States

**Keywords:** cognitive impairment, multiple sclerosis, diffusion MRI, brain networks, imaging bio-markers, Ollivier-Ricci curvature, brain networks robustness

## Abstract

The robustness of brain structural networks, estimated from diffusion MRI data, may be relevant to cognition. We investigate whether measures of network robustness, such as *Ollivier-Ricci curvature*, can explain cognitive impairment in multiple sclerosis (MS). We assessed whether local (i.e., cortical area) and/or global (i.e., whole brain) robustness, differs between cognitively impaired (MSCI) and non-impaired (MSNI) MS patients. Fifty patients, with Expanded Disability Status Scale mean (m): 3.2, disease duration m: 12 years, and age m: 40 years, were enrolled. Cognitive impairment scores were estimated from the Minimal Assessment of Cognitive Function in Multiple Sclerosis. Images were obtained in a 3T MRI using a diffusion protocol with a 2 min acquisition time. Brain structural networks were created using 333 cortical areas. Local and global robustness was estimated for each individual, and comparisons were performed between MSCI and MSNI patients. 31 MSCI and 10 MSNI patients were included in the analyses. Brain structural network robustness and centrality showed significant correlations with cognitive impairment. Measures of network robustness and centrality identified specific cortical areas relevant to MS-related cognitive impairment. These measures can be obtained on clinical scanners and are succinct yet accurate potential biomarkers of cognitive impairment.

## Introduction

The *robustness* of brain networks is defined as the “degree to which the topological properties of a network are resilient to lesions such as the removal of nodes or edges” (1). Removal of nodes (e.g., cortical or subcortical areas) and edges (e.g., white matter pathways) from such a network are computational constructs that model the disruptive effects of lesions or degeneration caused by trauma or disease. Brain functionality is often remarkably robust (*via* brain plasticity) to such lesions, as it adapts to the damage. However, it may be fragile and unable to adapt when the damage takes place at certain vulnerable locations in a specific amount, resulting in dysfunction or cognitive impairment (CI). Studies quantifying brain robustness, as it relates to impairment and dysfunction, can be broadly divided into two categories. First, those that use structure-to-function relationships to predict functional resilience to lesions in a given location (2). Second, those applying recently proposed geometric methods, based on the concept of graph *Ollivier-Ricci curvature* (referred to as curvature here onwards), which can quantify brain robustness and relate it to changes caused by healthy aging or brain disorders (3).

CI is observed in 40–65% of MS patients (4), affecting their quality of life and professional performance. CI in MS has been associated with structural disconnection due to demyelination and axonal damage (5). Diffusion MRI (dMRI), an effective imaging modality to study such neurodegeneration, can be used to build structural networks and describe connectivity changes during the course of the disease. In line with other brain robustness studies, several investigations of MS-related CI have used structural networks to explain cognitive dysfunction (5–8). Global and local network metrics show that in MS, information flow between brain regions is reduced (5, 6) causing dysfunction. At the whole brain level, global network metrics like network density and global efficiency capture topological changes related to impairment and provide valuable information complementary to clinical and atrophy measures (7). Changes in global efficiency of the structural network associated to the default mode network (DMN) by resting-state fMRI, were also related to CI in MS (9).

In order to improve the ability to detect differences between structural networks from healthy controls (HC) and MS patients, some studies have combined network metrics with machine learning approaches (10, 11). They use a wide range of global and local network metrics as inputs to train classifiers. However, the accuracy of these classifiers remains relatively low at 60–65% (10, 11), which can be mainly attributed to the low discriminatory power of many networks' metrics. Furthermore, a recent study on the effects of “edge deletion” in brain networks (modeling disconnection due to lesions) concluded that such networks tend to be robust to damages caused by MS lesions, and thus do not provide sensitive markers (8). Therefore, it is currently challenging to accurately categorize brain structural networks from healthy individuals or from people with MS. Consequently, *there is a crucial need to improve these networks' analysis techniques in order to (1) better characterize the effects of MS on brain networks, and (2) develop potential biomarkers for future clinical trials*.

This study leverages various brain networks measures, including the application of curvature (3) for the first time, to present a simple framework for identifying critical brain areas in MS related CI. Curvature positively correlates with the robustness of a network (3) and quantifies the overall brain functionality impact due to MS related lesions, which is not captured by traditional network analysis (3). At a global level, higher curvature (showing relative network stability) should indicate less CI. The idea is to use standard resolution clinical scans for generating dMRI-based macro networks, and instead of seeding in affected areas, randomly seed the entire brain for tractography. Also, for robustness analysis, instead of edge deletion modeling (8), we use curvature and network robustness/centrality measures to identify areas related to CI in MS. This makes our proposed approach more general, which can be readily applied across different scanners using diverse scanning protocols.

## Materials and Methods

50 MS patients, 10 cognitively non-impaired (MSNI) and 31 cognitively impaired (MSCI) participated in this study. Out of the excluded 9 patients, 5 were borderline CI and 5 were not scanned for dMRI and/or had missing clinical data. Detailed demographics of the cohort (given as Supplementary Note 1) and exclusion criteria can be found in our previous work (12). Written informed consent was obtained from each subject following University of Texas, Houston, TX, Institutional Review Board approval of the research protocol (12).

### Cognitive Assessment

To quantify cognitive function with psychometric testing, the Minimal Assessment of Cognitive Function in Multiple Sclerosis (MACFIMS) was used (13). Twenty MACFIMS parameters related to MS cognitive deficits were identified, and an overall CI index was derived. Patients were classified as MSNI if their performance was more than 1 standard deviation below the mean for 8 out of 20 parameters (40%), which corresponds to an CI index <0.2. Similarly, patients were classified as MSCI if they performed more than one standard deviation on 40% parameters, which corresponds to an CI index >0.35. Patients with score between 0.2 and 0.35 were considered as borderline and excluded from analysis.

### Diffusion MRI Data Acquisition and Pre-processing

MRI data were acquired on a Philips 3.0 T Intera scanner using a SENSE receive head coil as previously described (12). The dMRI data were acquired axially using a single-shot multi-slice 2-dimensional spin-echo diffusion sensitized and fat-suppressed echo planar imaging (EPI) sequence, with the balanced *Icosa21* tensor encoding scheme (21 unique directions). The *b*-factor was 1,000 s mm^−2^, TR/TE = 7,100/65 ms, FOV = 256 × 256 mm, and slice thickness/gap/number of slices = 3 mm/0 mm/44. The EPI phase encoding used a SENSE *k*-space under-sampling factor of 2, with an effective *k*-space matrix of 128 × 128, and an image matrix after zero-filling of 256 × 256. The reconstructed image spatial resolution was 1 × 1 × 3 mm. Acquisition time was ~2 min.

Scanner images (in DICOM format) were converted to NIFTI using dcm2nii (14) software. Eddy current distortion correction was applied using FSL eddy_correct (15) and the Brain Extraction Toolbox was used to mask out the brain (15).

### Brain Structural Networks

In previous studies, the following three types of structural networks have been used to analyze MS-related changes:

#### Tractography-Based Networks (7, 10)

These are region of interest (ROI) based connectivity maps of the entire brain. A diffusion tensor or multi-fiber model is fit to the diffusion data at each voxel to estimate the principal fiber orientation(s). Tractography is subsequently performed using these directions, and the connectivity (the network's edges) of cortical/subcortical ROIs (the network's nodes) is estimated as the number of fiber tracts between each pair of ROIs.

#### Fractional Anisotropy (FA)-Based Correlation Matrices (5, 6, 8)

The average FA values are estimated for each ROI. The values for all the participants in a cohort are then used to calculate the Pearson correlation coefficient between ROIs. The correlations values form a graph on a group level (e.g., MSNI).

#### Cortical Thickness-Based Correlation Matrices (16)

The average cortical thickness is calculated for each ROI. Instead of FA values as described above, correlations are calculated between each pair of ROIs to construct a graph on a group level.

We note that methods 2.3.2 and 2.3.3 above do not yield connectivity networks *per se*, but rather graphs/matrices which encode macro/microstructural similarities of brain areas. Therefore, we only compute tractography-based connectivity networks due to the following reasons: (1) The clustering coefficient, an important *measure of graph robustness*, can lead to incorrect inferences (17) in correlation networks and (2) Both FA and cortical thickness correlation matrices are inherently obtained at group level, which does not explicitly allow individual level statistical analysis within each cohort.

### Structural Connectivity Networks Construction

To generate connectivity matrices, diffusion tensors were estimated to perform tractography. In order to capture connectivity alterations induced by MS pathology at the network level, we chose to seed tractography randomly throughout the whole brain, contrary to Solana et al. (10). DSI Studio (18) was used to generate the connectivity matrices with the following settings: FA threshold = 0.1, angular threshold = 60°, tractography method: Runge–Kutta, total number of streamlines: 500,000. A total of 333 cortical areas (nodes) were automatically segmented *via* non-linear registration of the Gordon cortical template (19) available in DSI Studio. Connectivity matrices were constructed with weights defined as the number of streamlines connecting each pair of cortical areas. Node numbers (IDs), centroid and functional community (group) for each of the 333 cortical areas obtained from resting-state fMRI can be downloaded from https://sites.wustl.edu/petersenschlaggarlab/parcels-19cwpgu/. Local (node level) and global network measures were calculated using the Brain Connectivity Toolbox (https://sites.google.com/site/bctnet/), except for curvature which was calculated using our code, available at https://www.cmrr.umn.edu/downloads/gcurve/.

### Network Nodal Measures

From our previous work (3), we selected four local measures of network robustness: curvature, strength, betweenness centrality, and clustering coefficient. A brief description of these measures is given below, more details can be found in Farooq et al. (3).

#### Curvature

It is a direct measure of network robustness. A large curvature means a fast return to the original state after perturbation, while a small curvature corresponds to a slow return (fragility). Curvature is a local property that explains the contribution of each individual node to the overall brain network robustness (3).

#### Strength

Strength is the number of edges connecting a node to the rest of the network. It is the total weight of connections coming in and out of a node. Generally, the more connected a node is, the more central it is in the network. However, more connectedness may not always make a node more central, if it is not involved in the most efficient paths in the network.

#### Betweenness Centrality

It is a measure of centrality based on the number of shortest paths routing through the node (20). In a network, central nodes tend to be part of shortest paths.

#### Clustering Coefficient

It is the probability, for a given node, that all its neighboring nodes are also connected to each other. The clustering coefficient of a node ranges between 0 and 1. Clustering coefficient is helpful in assessing small-worldness of networks (17).

A brief description of the global measures of network robustness used in this study is provided in Supplementary Note 2.

### Statistical and Correlation Analysis of Network Measures and CI Scores

For comparisons between MSCI and MSNI structural networks at the cortical areas (nodes) level, corrections for multiple comparisons and family-wise error rate was controlled using the Holm–Sidak method with α = 0.05. The correction was performed using GraphPad Prism (https://www.graphpad.com/), with homoscedasticity assumption that is, data was sampled from normal distributions with identical standard deviations, while computing two-tailed unpaired *p*-values. The number of unpaired *t*-tests to correct for was equal to the number of nodes i.e., 333 for the Gordon atlas.

At global (whole brain) level, we hypothesize that network robustness measures will be higher in MSNI as compared to MSCI. For comparison, we perform un-paired one-tailed *t*-test corrected for multiple comparisons with α = 0.05.

To associate cognitive ability with network measures, we did not perform stepwise regression as done in some previous studies (7, 21). It is important to note that stepwise regression may be accurate when the number of “true” predictor variables is roughly equal to the number of “nuisance” variables (22), which is not the case while relating many network (or clinical) measures to cognitive ability/score. Resultant stepwise regression models may fit the data well for the samples under study, but perform poorly on independent datasets or out-of-sample validation (22).

## Results

### Local Brain Network Measures

**Figure 1:**
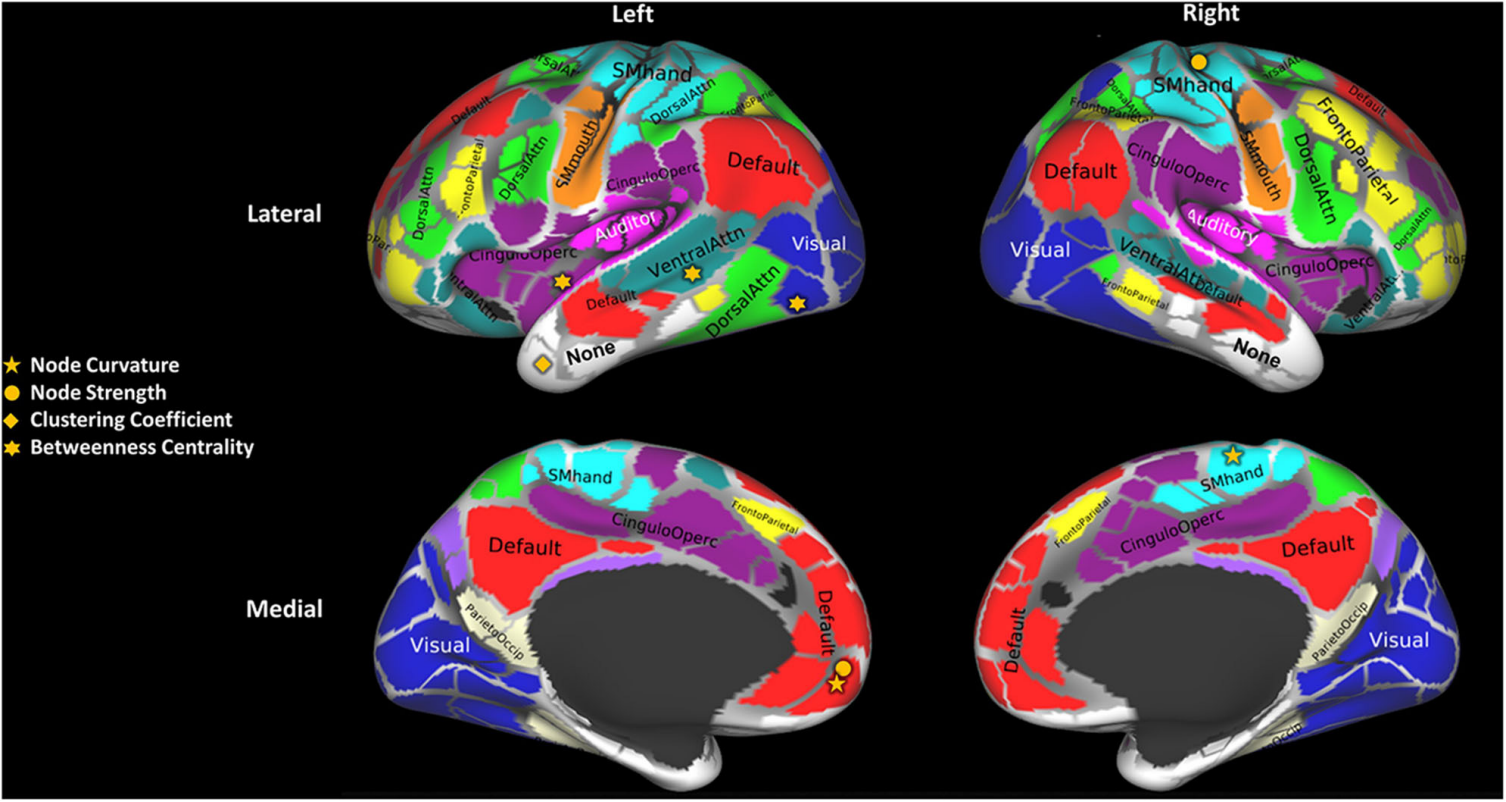
Cortical areas (network nodes) with statistically significant differences (corrected for multiple comparisons using the Holm–Sidak method) between MS cognitively impaired (MSCI) and non-impaired (MSNI) patients. Brain parcellation with 333 cortical areas was obtained using the Gordon atlas (19) and labeled with the Brain Analysis Library of Spatial maps and Atlases database https://balsa.wustl.edu/WK71. Adapted from Figure. 10 of Supplementary Data from Gordon et al. (19).

**Figure 2:**
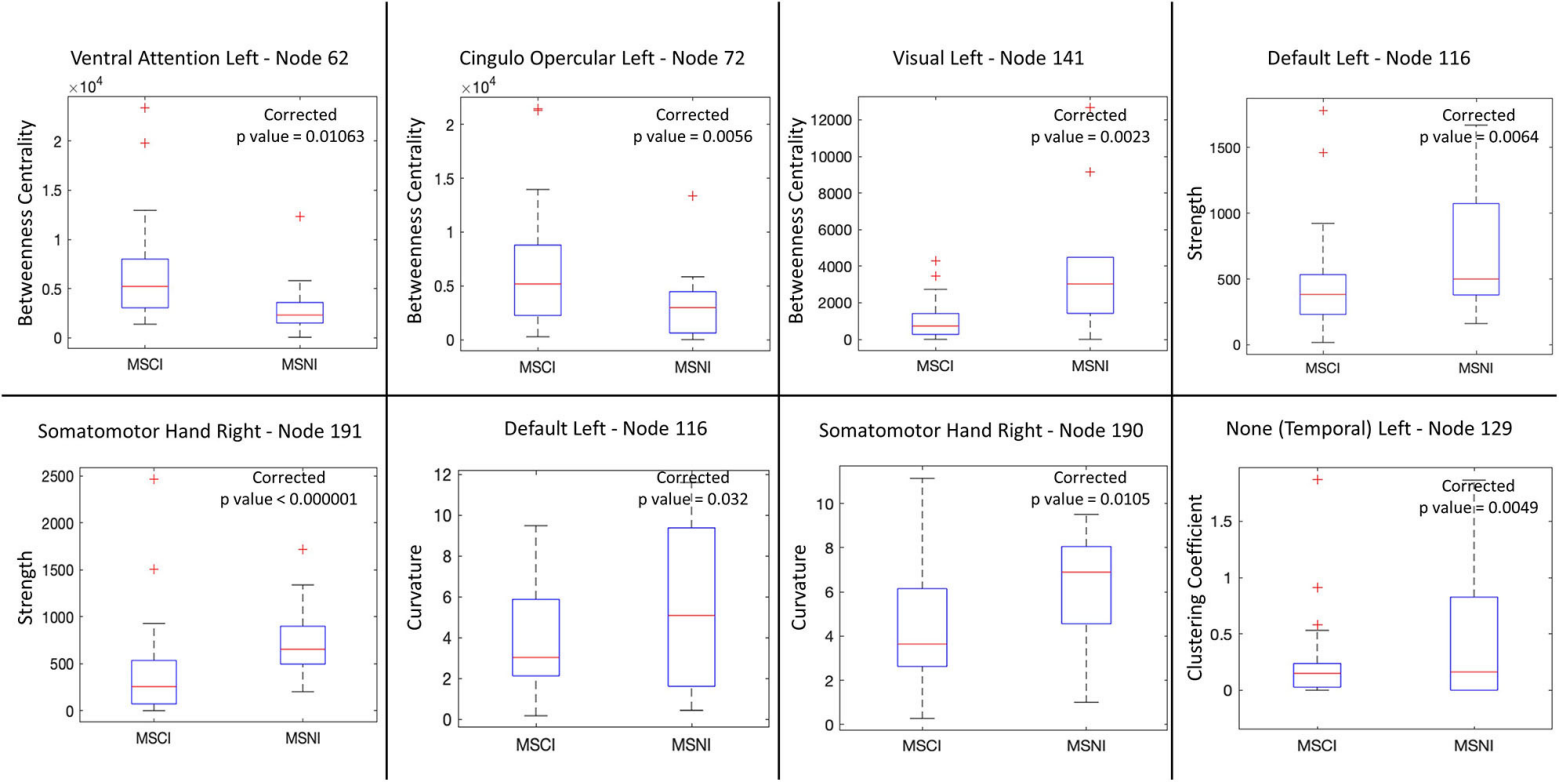
Differences in network (graph) local measures between MS cognitively impaired (MSCI) and non-impaired (MSNI) patients. Betweenness centrality is increased in nodes from the ventral attention and cingulo-opercular networks in MSCI patients. For the other nodes, MSCI show decrease in all measures.

Local measures identified significant structural connectivity differences between MSCI and MSNI patients in six cortical areas. We noted a left asymmetry for these areas, as can be seen in Figure 1. The areas include parts of the ventral attention, cingulo-opercular, default mode, temporal and visual networks in the left hemisphere, and somato-motor (hand) network in the right hemisphere (Supplementary Note 3). Figure 2 shows the overall change in local measures between MSCI and MSNI. Betweenness centrality is found to be increased in cortical areas that are part of the ventral attention and cingulo-opercular networks in MSCI, compared to MSNI, and decreased in cortical areas that are part of the visual network. Curvature and strength show similar results of overall decrease in parts of the default mode and somato-motor networks in MSCI patients. In temporal areas, the clustering coefficient is decreased in MSCI patients.

### Global Brain Network Measures

At the global network level, clustering coefficient, curvature, density, diameter, efficiency, characteristic path length, and small worldness were compared. As expected, MSNI cohort showed statistically significant higher curvature. However, no other global measure captured any significant difference between the groups.

## Discussion

In this pilot study, we provide curvature-based brain networks analysis of structural changes between MSNI and MSCI patients, to evaluate their potential as imaging biomarkers for CI. We used network centrality and robustness measures to detect areas related to CI and present a simple framework which is clinically feasible from scanning time and computational efficiency's stand points.

### Local Brain Network Measures

#### Left Hemispheric Asymmetry of Differences Related to CI

In MS, the dominant hemisphere may have increased vulnerability to the pathological processes, and thus an asymmetric interhemispheric lesion distribution (23) as well as structural damage (24). Further, a longitudinal study focusing on regional patterns of focal lesions accumulation and tissue atrophy progression (24) associates left-lateralized pattern of gray matter and white matter atrophy to cognitive and clinical deterioration. We found a consistently asymmetric pattern using local network measures as shown in Figure 1.

#### Ventral Attention Areas

Functional connectivity studies show that, in MS patients, there is a likelihood of increased activation in the left ventrolateral pre-frontal cortex inducing increased activation of the ventral attention network as compared to HC (25). In our study, MSCI patients, showed increased betweenness centrality for the structural connectivity of left ventral attention areas, compared to MSNI, which may be related to the reported increased activation of those areas. In MSCI, those ventral attention areas route more “information” and become more central in overall brain functionality.

#### Cingulo-Opercular Areas

The cingulo-opercular functional network is thought to support stable maintenance of task and strategy during cognitive processes (26). It forms part of the cognitive control network and functions to maintain tonic alertness (27). In MSCI patients, increased centrality in the anterior insula/frontal operculum (part of the salience network) was found (Figure 1). Similar to the ventral attention areas, this may be explained as a compensatory mechanism/brain plasticity, making the region structurally more pivotal. This conforms to findings of increased functional activity in MS patients in the frontoparietal network and the thalamus (part of the cingulo-opercular network) (28).

#### Visual Areas

Visual impairment is an important manifestation of MS, found in both structural and resting-state fMRI analysis (29). In addition to an overall decrease in the activation of the primary visual cortex, a specific decrease in the functional connectivity of left hemisphere visual areas in MS patients (30) is known, based on resting-state fMRI analysis. Consistent with these studies, we find decreased betweenness centrality of structural connectivity in the visual areas of MSCI patients, which may be related to damaged pathways modifying the overall visual structural network despite adequate visual acuity of all patients enrolled.

#### DMN

The DMN's reduced functional connectivity is related to CI in MS (31). Also, DMN's structural global efficiency is reduced in CI (9) and atrophy of the frontal cortex can predict CI in MS (32). In line with these studies, we find that the frontal areas of the DMN show reduced node strength and node curvature in MSCI patients. Reduced node strength indicates loss of tracts to and from the ventromedial pre-frontal cortex area. Reduced nodal curvature implies reduced contribution of this area to the overall stability of the DMN.

#### Somato-Motor (Hand) Areas

Studies using fMRI have shown decreased functional connectivity in the sensorimotor network in MS, which correlates with motor disability (33). In this study, both node strength and curvature found distinct nodes in the somato-motor area with decreased values in MSCI, suggesting decreased structural connectivity. Decreased local curvature indicates that the area is contributing to the overall vulnerability of the brain network in MSCI patients.

#### Temporal Areas

The temporal lobe is involved in memory function and information processing speed. In MS, atrophy of the right temporal lobe has been suggested to correlate with CI (34). In resting-state functional networks, formation of hubs in the left temporal lobe are found in MS patients which are not present in HC (35). Here, a decreased clustering coefficient of structural networks in the left temporal area of MSCI patients implies that the community structure is less pronounced, affecting the temporal lobe functionality and contributing to MS related impairment (17).

### Global Results

Global curvature shows significant decrease in MSCI and can therefore be hypothesized as the most sensitive measure to CI (Supplementary Figure 1). This result is expected because brain networks in MSNI patients may be more robust in functionality. All other global measures did not show significant differences between groups.

### Correlations of Network Measures and CI Index

**Figure 3:**
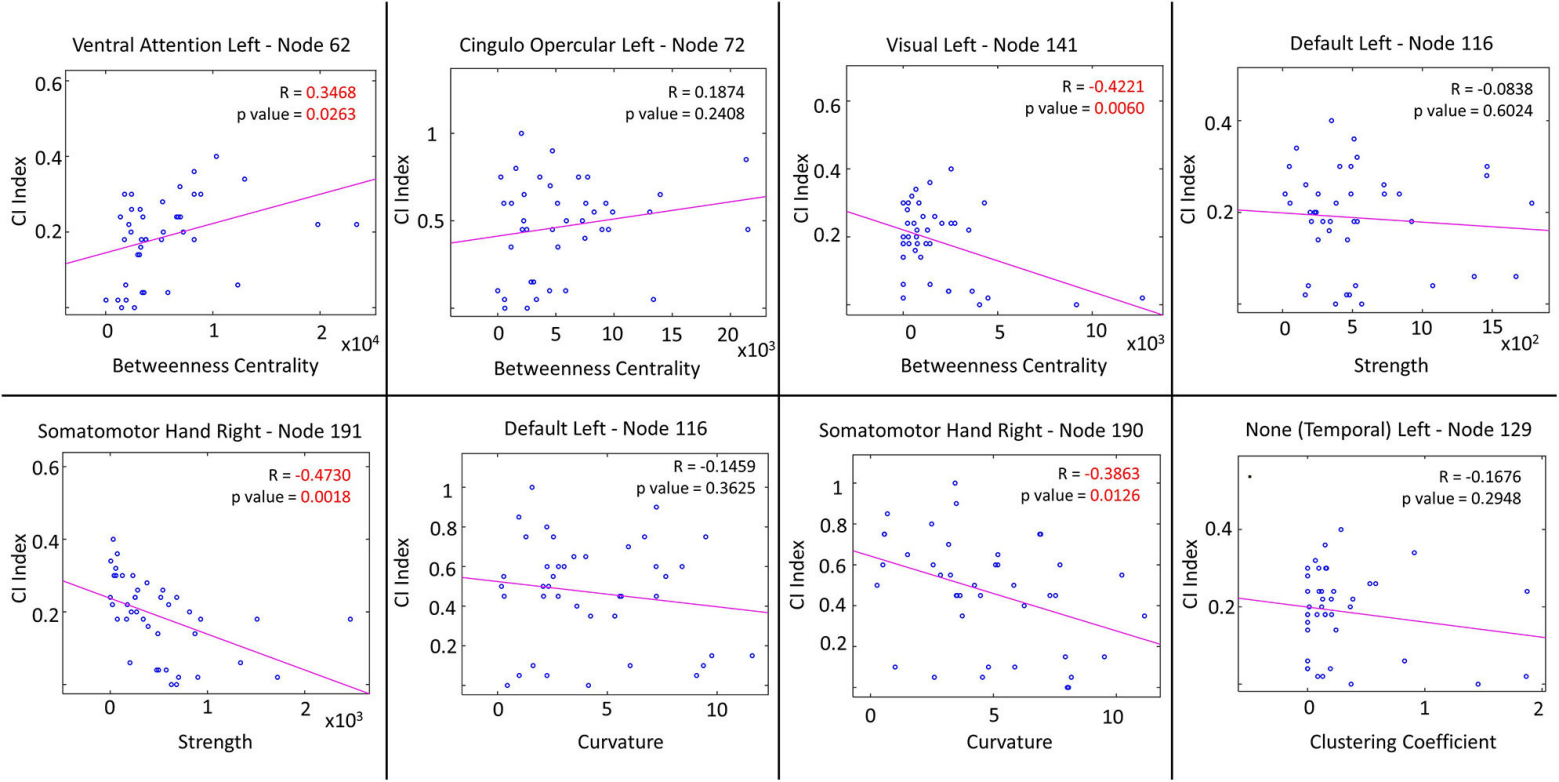
Correlation plots between cognitive impairment (CI) index and network local measures, for brain areas showing significant differences between MSCI and MSNI patients. Plots with significant *p*-values (shown in red) show positive correlation between CI and betweenness centrality in a node from the ventral attention network. A negative correlation between CI and betweenness centrality in a node from the visual network, and with strength as well as with curvature in nodes from the somatomotor (hand) network is observed.

Local cortical areas showing significant differences between cohorts were investigated for possible correlation between network measures and CI index as shown in Figure 3. Betweenness centrality is positively correlated with CI index in the left ventral attention areas, and negatively correlated with CI index in the left visual areas. Similarly, both strength and curvature are negatively correlated with CI index in the somato-motor areas. Statistically significant correlation values are shown in red in Figure 3. Only global curvature significantly correlates (negatively) with CI index (Supplementary Figure 2).

This study should be considered in view of some limitations. First, the sample size of MSNI and MSCI cohorts is small and not ideally balanced in size/power. Second, being cross-sectional, it cannot accurately describe progressive structural changes due to CI in MS. Further longitudinal studies with large sample size are required to study CI in MS using graph-theoretical methods.

## Conclusion

Brain networks robustness and centrality measures provide invaluable information about CI in MS patients. Based on this exploratory study, we conclude that curvature, both at the local and global level, accurately classifies MSCI/MSNI patients and correlates with CI index. These measures, shown to be powerful tools to study network fragility, may be valuable to better characterize MS related CI, especially in combination with neural networks/compact prediction models, as compared to the ones used in previous studies (10). However, studies with larger cohort sizes, as well as longitudinal studies, are required to further ascertain the sensitivity of network robustness measures to MS related CI.

## Data Availability Statement

Although raw diffusion dataset cannot be made available, connectivity matrices can be provided on request to the corresponding author, Hamza Farooq, faroo014@umn.edu.

## Ethics Statement

The studies involving human participants were reviewed and approved by Institutional Review Board (Study Number HSC-MS-10-0693), The University of Texas, Houston, United States. The patients/participants provided their written informed consent to participate in this study.

## Author Contributions

HF, CL, and FN conceived and planned the experiments. FN carried out the data acquisition. HF wrote the manuscript and conducted the experiments. CL provided critical feedback on the results and the manuscript.

## Conflict of Interest

The authors declare that the research was conducted in the absence of any commercial or financial relationships that could be construed as a potential conflict of interest.
