# Supplementary material for: Robustness of Brain Structural Networks Is Affected in Cognitively Impaired MS Patients
Source: Front Neurol. 2020 Nov 19;11:606478. doi: 10.3389/fneur.2020.606478 (PMC7710804; doi:10.3389/fneur.2020.606478)
Supplement: Supplementary file 1 [file Data_Sheet_1.docx]

Supplementary Material

**Supplementary Note 1 – Demographics**

|  | Cognitively Non-Impaired  (MSNI) | Cognitively Impaired  (MSCI) |
| --- | --- | --- |
| Number of subjects | 10 | 31 |
| Age | 36.7 | 42.3 ± 11.33 |
| Gender | 6 Female, 4 Male | 25 Female, 6 Male |
| Disease duration (years) | 9.65 ± 8.19 | 14.6129 ± 9.7559 |
| Expanded Disability Status Scale (EDSS) | 1.3 ± .78 | 4.3064 ± 1.8059 |
| Cognitive Impairment Index | 0.075 ± 0.054 (range from 0 to 0.25) | 0.5967 ± 0.1627 (range from 0.35 to 1) |
| MS subtypes | 10 Relapsing-Remitting | 23 Relapsing-Remitting, 7 Secondary Progressive and 1 Primary Progressive |

Mean ± standard deviation for age, disease duration, EDSS and cognitive impairment index.

**Supplementary Note 2 - Network Global Measures**:

*Global graph curvature*:

It is the average nodal curvature, measuring on average how robust a network is to lesions(1, 2).

*Small worldness*:

It is a measure of balance between network segregation and integration, defined as the combination of high clustering and short characteristic path length. In other words, it is a combination of high global and local efficiency of information transfer between nodes of a network(3).

*Global efficiency*:

It is a measure of information exchange efficiency for the whole network, described as the average of the inverse shortest path length from a given node to all other nodes(4).

*Diameter:*

It is the length of the shortest path between the most distanced nodes of a graph. It measures the extent of a graph and the topological length between two nodes.

*Density:*

It is the fraction of existing vs. actual connections to possible connections in a network. (<https://www.the-vital-edge.com/what-is-network-density/>)

*Clustering coefficient:*

It is the average of the clustering coefficients of all individual nodes of the network. The clustering coefficient is considered to be a measure of local connectivity of a graph. High clustering is associated with resilience against random network damage. (<https://home.kpn.nl/stam7883/graph_introduction.html>)

*Characteristic path length:*

It is the average distance from a node to all other nodes, for all the nodes of a given network. At the global level, the average of the path lengths of all nodes provides the characteristic path length (<http://braph.org/manual/graph-measures/>).

**Supplementary Note 3 – Characteristics of statistically different brain areas (nodes) between MSCI and MSNI patients**:

| Node Number (parcel id) | Hemisphere | Centroid (MNI) | Functional Community | Corresponding Brodmann Area |
| --- | --- | --- | --- | --- |
| 62 | Left | -48.1 -40 2.4 | Ventral attention | Middle temporal area (BA 21) |
| 72 | Left | -39.1 -1.6 -12.2 | Cingulo-opercular | Insula (BA 13) |
| 116 | Left | -5.9 54.8 -11.3 | Default (Default Mode Network) | Anterior frontal cortex (BA 10) |
| 129 | Left | -44.6 9 -37 | None (Temporal) | Temporal pole (BA 38) |
| 141 | Left | -22.6 -81.7 -11.7 | Visual | Visual Association Area (BA 18) |
| 190 | Right | 16.5 -32.8 67.7 | Somato-motor (hand) | Primary somatosensory (BA 1) |
| 191 | Right | 4.8 -27.1 64.8 | Somato-motor (hand) | Primary somatosensory (BA 4) |

**Supplementary Figures**


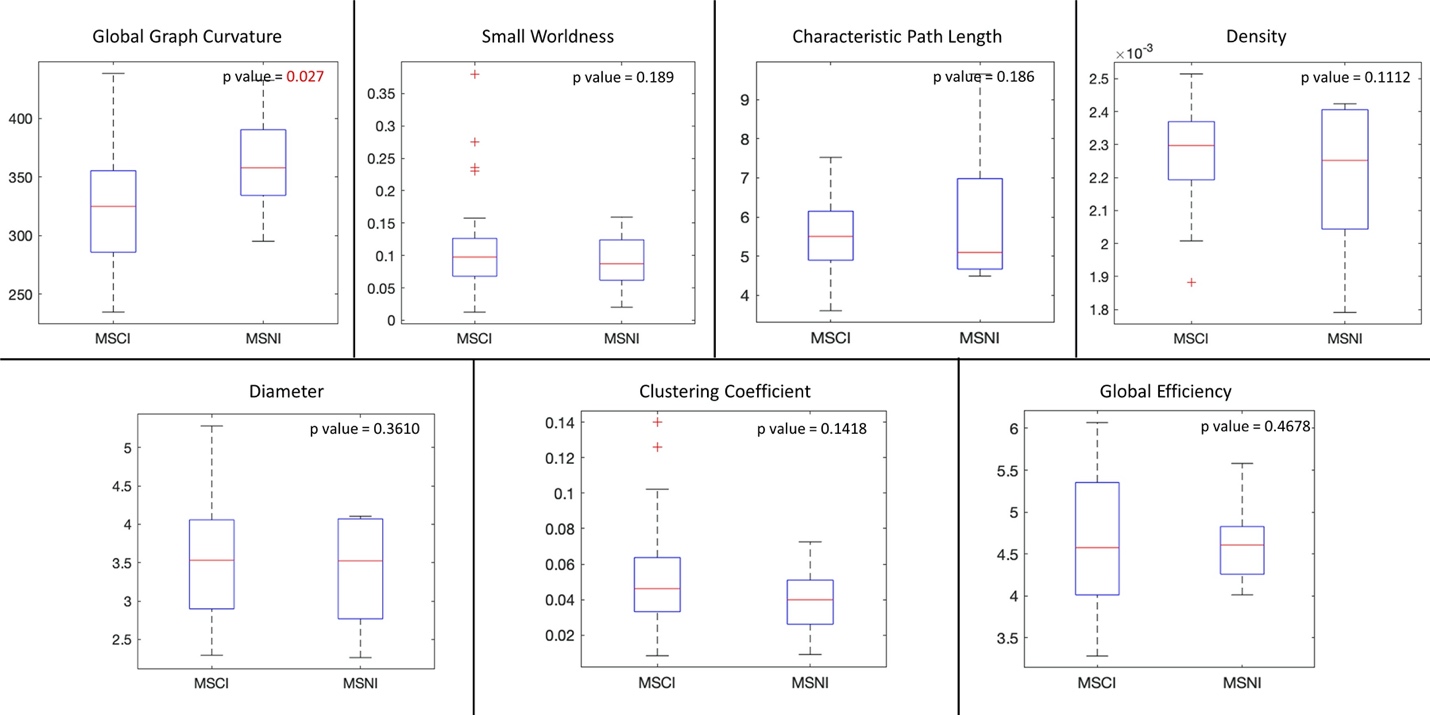


**Supplementary Figure 1.** Difference in global network measures between MS cognitively impaired (MSCI) and non-impaired (MSNI) patients. Global graph curvature is the only measure showing significant decrease in the MSCI group.


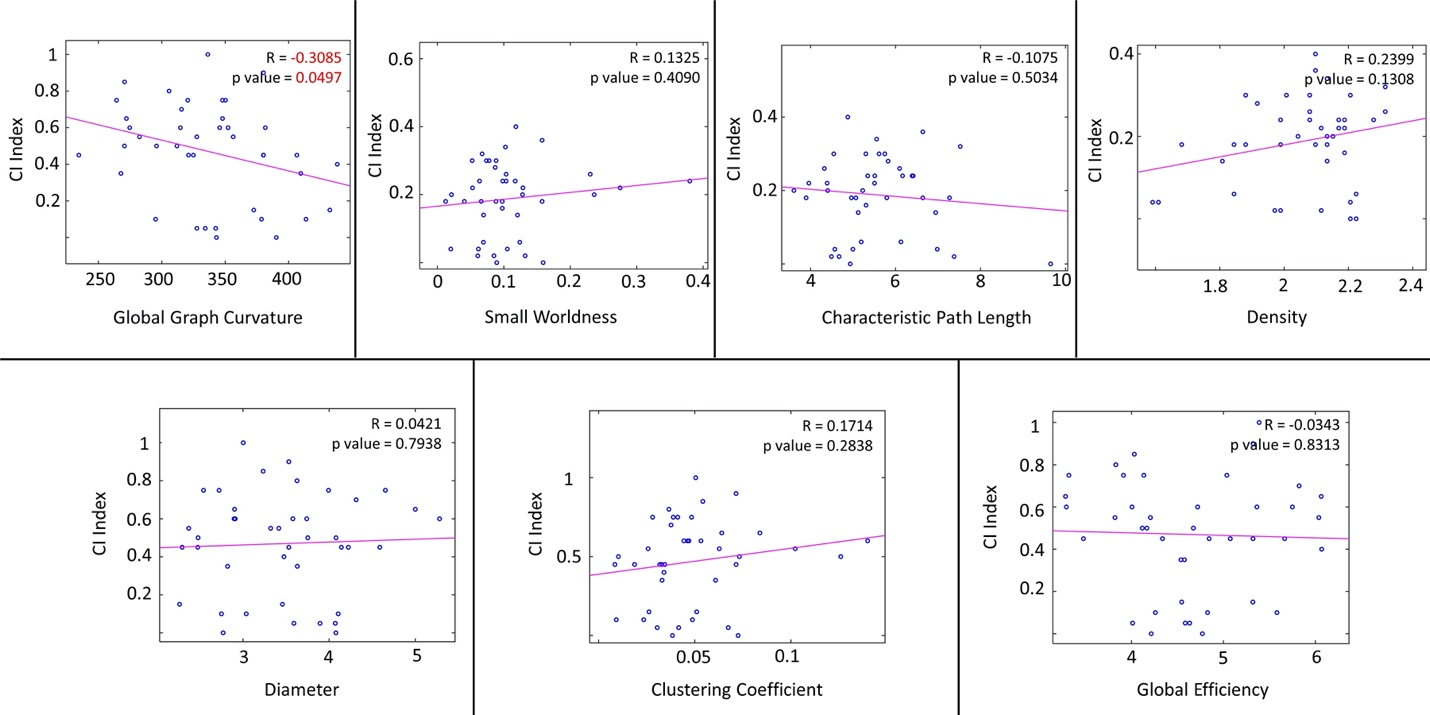


**Supplementary Figure 2**.Correlation plots between the cognitive impairment (CI) index and global graph measures. Global graph curvature shows negative correlation with the CI Index, meaning that higher impairment correlates with overall fragility of the brain network.

**References:**

1. Sandhu RS, Georgiou TT, Tannenbaum AR. Ricci curvature: An economic indicator for market fragility and systemic risk. *Science Advances* (2016) 2(5). doi: 10.1126/sciadv.1501495. PubMed PMID: WOS:000380073000008.

2. Farooq H, Chen YX, Georgiou TT, Tannenbaum A, Lenglet C. Network curvature as a hallmark of brain structural connectivity. *Nature Communications* (2019) 10. doi: 10.1038/s41467-019-12915-x. PubMed PMID: WOS:000493275600011.

3. Bullmore ET, Bassett DS. Brain Graphs: Graphical Models of the Human Brain Connectome. *Annual Review of Clinical Psychology* (2011) 7:113-40. doi: 10.1146/annurev-clinpsy-040510-143934. PubMed PMID: WOS:000290238600005.

4. Latora V, Marchiori M. Efficient behavior of small-world networks. *Physical Review Letters* (2001) 87(19). doi: 10.1103/PhysRevLett.87.198701. PubMed PMID: WOS:000172027200063.
